# Abdominal Fat Characteristics and Mortality in Rectal Cancer: A Retrospective Study

**DOI:** 10.3390/nu15020374

**Published:** 2023-01-11

**Authors:** Massimo Pellegrini, Giulia Besutti, Marta Ottone, Simone Canovi, Efrem Bonelli, Francesco Venturelli, Roberto Farì, Angela Damato, Candida Bonelli, Carmine Pinto, Guido Ligabue, Pierpaolo Pattacini, Paolo Giorgi Rossi, Marwan El Ghoch

**Affiliations:** 1 Department of Biomedical, Metabolic and Neural Sciences, University of Modena and Reggio Emilia, 41121 Modena, Italy; 2 Radiology Unit, Azienda USL–IRCCS di Reggio Emilia, 42123 Reggio Emilia, Italy; 3 Department of Medical and Surgical Sciences, University of Modena and Reggio Emilia, 41121 Modena, Italy; 4 Epidemiology Unit, Azienda USL–IRCCS di Reggio Emilia, 42123 Reggio Emilia, Italy; 5 Clinical Chemistry and Endocrinology Laboratory, Azienda USL–IRCCS di Reggio Emilia, 42123 Reggio Emilia, Italy; 6 Oncology Department, Azienda USL–IRCCS di Reggio Emilia, 42123 Reggio Emilia, Italy; 7 Department of Nutrition and Dietetics, Faculty of Health Sciences, Beirut Arab University, Riad El Solh, Beirut 11072809, Lebanon

**Keywords:** adipose tissue, computed tomography, rectal cancer, body composition, survival

## Abstract

The aim of this study was to evaluate the association of adipose tissue characteristics with survival in rectal cancer patients. All consecutive patients, diagnosed with stage II–IV rectal cancer between 2010–2016 using baseline unenhanced Computed Tomography (CT), were included. Baseline total, subcutaneous and visceral adipose tissue areas (TAT, SAT, VAT) and densities (TATd, SATd, VATd) at third lumbar vertebra (L3) were retrospectively measured. The association of these tissues with cancer-specific and progression-free survival (CCS, PFS) was assessed by using competitive risk models adjusted by age, sex and stage. Among the 274 included patients (median age 70 years, 41.2% females), the protective effect of increasing adipose tissue area on survival could be due to random fluctuations (e.g., sub-distribution hazard ratio—SHR for one cm^2^ increase in SAT = 0.997; 95%confidence interval—CI = 0.994–1.000; *p* = 0.057, for CSS), while increasing density was associated with poorer survival (e.g., SHR for one Hounsfield Unit—HU increase in SATd = 1.03, 95% CI = 1.01–1.05, *p* = 0.002, for CSS). In models considering each adipose tissue area and respective density, the association with CSS tended to disappear for areas, while it did not change for TATd and SATd. No association was found with PFS. In conclusion, adipose tissue density influenced survival in rectal cancer patients, raising awareness on a routinely measurable variable that requires more research efforts.

## 1. Introduction

The link between body composition and health outcomes has gained interest in several clinical settings, as well as among the general population [1,2,3]. Specifically, in recent years, there has been a particular interest in body composition variables, which can predict oncological outcomes [4], with the primary aim of improving treatment personalization and consequently, the survival of patients diagnosed with cancer [5].

Firstly, studies on body composition and cancer have primarily focused on anthropometric measures and indices of muscle and fat quantity. The higher cancer risk in patients with higher body mass index (BMI) is well known for many cancer types, including colorectal cancer (CRC) [6,7]. On the other hand, the relationship between BMI and cancer mortality has been described as a U-shaped or J-shaped association, suggesting that both cachexia and obesity are associated with poor prognosis in most cancer types [8,9,10]. Studies evaluating the potential role of computed tomography (CT)-derived biomarkers of skeletal muscle quantity, as well as adipose tissue quantity in different fat compartments, have shown poorer outcomes in patients with higher visceral obesity and reduced skeletal muscle index in various different cancers, including CRC [11].

Over the past few years, besides the quantities of fat and muscle, there has been a focus on the study of the radiodensity of skeletal muscle mass and adipose tissue measured by CT as new prognostic biomarkers of oncological outcomes. For instance, a high adipose tissue radiodensity has been found to be associated with lower survival rates among patients with oesophageal cancer [12], pancreatic adenocarcinoma [13], hepatocellular carcinoma [14], head-and-neck cancer [15], extremity sarcoma [16] and multiple myeloma [17], as well as with other poorer outcomes such as increased post-surgical infection and tumour recurrence in patients with soft tissue sarcoma [18].

Few studies are available on the prognostic utility of CT tissue density assessments in CRC patients. Two studies found that the low radiodensity of the muscle mass (i.e., higher fat content within muscle fibres) was associated with lower survival rates [19,20], while another study did not find a significant association after adjusting for other prognostic factors [21]. Only one study investigated the prognostic role of adipose tissue radiodensity in CRC patients, showing lower survival rates among patients with higher fat density (this may reflect both tissue inflammation and/or smaller or shrunken adipocytes, according to different hypotheses) [22]. To the best of our knowledge, no previous study has been conducted focusing solely on patients with rectal cancer–a cancer with a significantly increased incidence rate among persons below the age of 50 living in Western countries (i.e., Finland, Australia, Canada and the United States) [23].

The aim of this study was to retrospectively evaluate the association between the adipose tissue area and radiodensity, and the survival rates of patients diagnosed with stage II–IV rectal cancer.

## 2. Materials and Methods

### 2.1. Study Design, Population and Ethics

This was a retrospective study including all consecutive patients diagnosed with stage II–IV rectal cancer between 2010 and 2016 in the Reggio Emilia province (Northern Italy, 530,000 inhabitants), after the exclusion of patients with an unavailable abdominal unenhanced CT scan performed at the time of diagnosis. Stage I rectal cancer patients were not included because baseline CT is not routinely recommended for staging small, low-risk lesions. The study was approved by the local ethics committee (Comitato Etico dell’Area Vasta Emilia Nord) with protocol number 2019/0079373. Given the retrospective nature of the cohort selection and data collection, the ethics committee authorized the use of a patient’s data without his/her informed consent after all reasonable efforts have been made to contact the patient to obtain it.

### 2.2. Clinical Data

Data on patients’ health status, rectal cancer diagnosis, staging, therapy and outcomes were extracted from the local population-based cancer registry, also drawing from the electronic medical records that are centralized for all the public hospitals and outpatient clinics within the provincial local health authority. CT images were retrieved from the radiology information system, namely, the picture archiving and communication system (RIS-PACS) of the local health authority.

### 2.3. Assessment of CT Adipose Tissue Characteristics

Staging and pre-treatment unenhanced CT images were retrospectively analyzed by a single trained operator (EB), supervised by a single radiologist (GB), both blinded to clinical data and outcomes, using the OSIRIX-Lite software V5.0 (Pixmeo, Sarl, Switzerland). A single slice at the level of the third lumbar vertebrae (L3), with both transverse processes visible, was selected. Abdominal fat compartments were selected after applying Hounsfield Unit (HU) thresholds ranging from −190 to −30 [24]. The total adipose tissue area (TAT), subcutaneous adipose tissue area (SAT), and visceral adipose tissue area (VAT) at the level of L3 were obtained through autosegmentation, and manual contour correction when necessary [25]; intra-organ fat (e.g., intrarenal adipose tissue) was manually excluded. The mean radiodensities were collected from the same regions of interest used for adipose tissue compartment areas (total adipose tissue density, TATd; subcutaneous adipose tissue density, SATd; visceral adipose tissue density, VATd) (Supplementary Figure S1). The VAT/SAT ratio was calculated as an indicator of adipose tissue distribution in different body fat compartments.

### 2.4. Outcome Measures

The two primary outcomes were defined as follows: (a) CSS was defined as the period from disease diagnosis to rectal cancer-related death; data were censored at the last medical evaluation; (b) PFS was defined as the period from diagnosis to first progression (locoregional or distant) or death from any cause, whichever came first, excluding stage IV or patients with missing data on progression.

Overall survival (OS), defined as the period from disease diagnosis to all-cause death, was considered as a secondary outcome.

### 2.5. Statistical Analyses

Clinical and CT data were reported as absolute frequencies and percentages for categorical variables and medians, and interquartile ranges were given for continuous variables. Spearman’s correlation was used to evaluate the associations between different adipose tissue CT parameters, in particular between areas and their respective densities. Mean CSS, OS and PFS survival times were computed and displayed through a survival curve using the Kaplan–Meier method. The unadjusted Cox proportional hazards model was used to verify the linearity of the association between CT body fat parameters and survival outcomes: according to this preliminary analysis, CT parameters were then introduced as continuous (if linearly associated with the outcome) or categorical (quartile) variables in subsequent survival models. The association between body fat characteristics and survival was assessed using the competitive risk model for CSS and PFS, according to the method of Fine and Gray [26], to estimate adjusted sub-distribution hazard ratio (SHR) with the corresponding 95% confidence intervals (CIs). Deaths from other causes were treated as competing events.

Models were adjusted for a set of covariates selected a priori: age at diagnosis (as a continuous variable), sex and tumour stage. In the case of CSS, survival analysis was modelled with and without the inclusion of stage IV patients, while for PFS, models excluding stage IV patients were computed. Secondarily, using models which include both adipose tissue areas and respective densities, we compared the point estimate and standard error of the coefficients, measuring the precision of the estimates. Additionally, we evaluated whether a component alone (area or density) may explain the outcome, independently from the other. Finally, similar models were built for CSS and PFS, including ones that used both SAT and VAT densities together.

In this study, sample size was determined by the number of available cases. Thus, no plans were made to detect it on the basis of a minimal clinically significant difference, nor any made to reach a statistical power with which to identify larger differences. Therefore, we did not fix any significance threshold to refuse or accept the null hypothesis; *p* values, as well as 95% confidence interval bounds, should be interpreted as an indication of the probability that the observed differences occurred under the null hypothesis without a pre-fixed threshold of significance.

Stata/IC 16.1 (Stata Corporation, College Station, TX, USA) software was used for the competing risk analysis (“stcrreg” command) and MedCalc 18.2.1 (MedCalc Software, Ostend, Belgium) was used for the other analyses.

## 3. Results

### 3.1. Study Population

**Figure 1:**
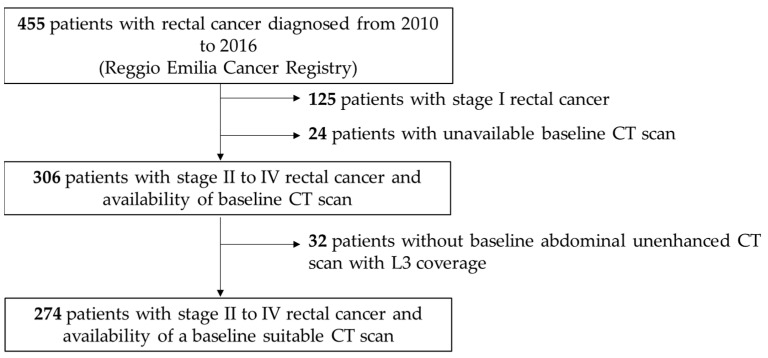
Flowchart representing patient inclusion. CT, Computed Tomography; L3, third lumbar vertebra.

After the exclusion of stage I rectal cancer, as well as patients with an unavailable or inadequate CT scan (Figure 1), we included 274 patients diagnosed with stage II–IV rectal cancer between 2010 and 2016. The median age at the time of diagnosis was 70 (59–79) years, and 113 (41.2%) patients were female. The stages at the time of diagnosis were stage II in 41 patients (15.0%), stage III in 161 patients (58.7%) and stage IV in 72 patients (26.3%), and the median BMI at the time of diagnosis was 21.2 Kg/m2 (19.0–23.8) (Table 1). The median follow-up for OS was 72 (52–95) months. The distribution of adipose tissue characteristics according to stage, age, and sex is reported in Supplementary Tables S1 and S2.

### 3.2. Survival

The mean OS was 65 (95% CI = 59–70) months and the mean CSS was 72 (95% CI = 66–78) months; 143 (52.2%) deaths were registered (116 cancer-specific, 25 from other causes and 2 from unknown cause). Excluding patients with stage IV disease, the mean PFS was 77 (95% CI = 70–84) months, and 63 (32.0%) events were registered.

### 3.3. Impact of Body Fat Characteristics on Survival

In a preliminary analysis (Supplementary Table S3), the association between body fat characteristics expressed in quartiles and outcomes was evaluated, with most parameters indicating a linear relationship with survival. The only exception was the relationship between TAT and OS, which was better represented by a model including quartiles, rather than a model including continuous variables.

As reported in Table 2, increasing visceral and subcutaneous adipose tissue areas had a protective effect on both CSS and PFS, more apparent in analyses excluding stage IV patients, although compatible with random fluctuations (type 1 error probability = 0.07). Similarly, for total adipose tissue area, the two lower quartiles had markedly lower survival rates than the two higher quartiles. On the contrary, increasing adipose tissue density was associated with lower survival rates in all analyses, with a stronger effect on CSS (SHR for one HU TAT increase = 1.033, 95% CI = 1.012–1.054, *p* = 0.002; SHR for one HU VAT increase = 1.02, 95% CI = 1.002–1.038, *p* = 0.027; SHR for one HU SAT increase = 1.03, 95% CI = 1.01–1.05, *p* = 0.002). No association was found between VAT/SAT ratio and survival.

**Figure 2:**
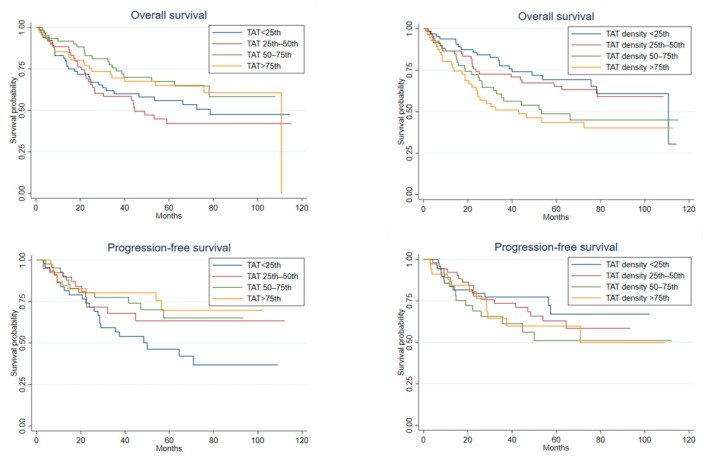
Kaplan–Meier survival curves for overall survival (*n* = 274) and progression-free survival (*n* = 197) by quartiles of total adipose tissue area (TAT) and total adipose tissue density (TAT density).

The graphs for the Kaplan–Meier survivor function for OS and PFS, indicated by quartiles of TAT area and density, are reported in Figure 2. Meanwhile Supplementary Figures S2 and S3, reports similar graphs by quartiles of VAT and SAT areas and densities.

### 3.4. Models including Both Density and Area

**Figure 3:**
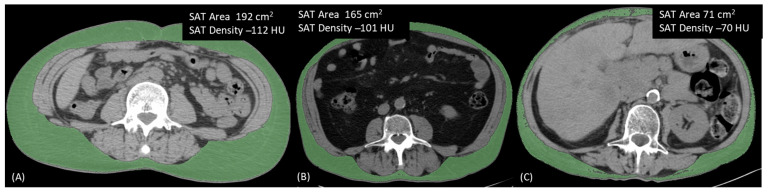
Three different patients illustrating the inverse relationship between area and density in a single adipose tissue compartment. (**A**) Patient with high SAT area and low SAT density; (**B**) Patient with both area and density showing intermediate values; (**C**) Patient with low SAT area and high SAT density. SAT, Subcutaneous Adipose Tissue.

Spearman’s correlations showed a strong inverse association between each adipose compartment area and its respective density (coeff = −0.7, *p* < 0.0001 for TAT and SAT, coeff = −0.8, *p* < 0.0001 for VAT) (Supplementary Figure S4, Figure 3). In competing risk models for CSS and PFS, including each adipose compartment area with its respective density, the hazard ratios for the adipose tissue area moved towards 1, while those between the adipose tissue density and CSS, especially in the case of TAT and SAT, did not change (SHR for one HU TAT increase = 1.03, 95% CI = 1.004–1.066, *p* = 0.028; SHR for one HU SAT increase = 1.03, 95% CI = 1.003–1.056, *p* = 0.026) (Table 3). Finally, a moderate positive correlation was found between SATd and VATd (Supplementary Figure S5), and in a model including both SATd and VATd together, only SATd retained an effect on CSS, although it was compatible with random fluctuations (type 1 error probability = 0.075) (Supplementary Table S4).

## 4. Discussion

Adipose tissue radiodensity, more than area, influenced survival rates among patients affected by stage II–IV rectal cancer. In fact, an increase in the radiodensity of TAT, SAT and VAT, assessed upon diagnosis, was associated with worse CSS over a follow-up period of nearly six years. This was true, even after adjustment for fat areas. This effect is consistent across models even if, at least for VAT, it is compatible with random fluctuations. The TAT area was associated with CSS, but the association almost disappeared after adjusting by the TAT density in the model. No association was found with PFS.

In general, our finding is in line with previous studies investigating several cancers (i.e., oesophagus, pancreas, liver, blood, soft tissue and head-and-neck) [12,13,14,15,16,17,18]. Of those, only one study was conducted on a population (i.e., CRC) similar to that investigated in our study [22], and examined the adipose tissue radiodensity prognostic utility, showing a linear association between VAT and SAT density and all-cause mortality in a large cohort of over 3000 patients.

The underlying mechanism behind this relationship is still unclear and needs clarification. Indeed, lower fat tissue density may reflect larger adipocytes filled by large lipid droplets (i.e., a potentially worst fat in terms of cardiometabolic risk) [27], while higher fat density may indicate a lower lipid content of adipocytes due to shrinkage and fibrosis (possibly following patient weight loss, a known poor prognostic factor at cancer diagnosis) [12] or inflammation of adipose tissue [24]. Moreover, since inflammation was found to be associated both with increased adipose tissue density and with reduced OS in patients with cancer cachexia [28], we cannot exclude the fact that inflammation can be a common denominator in this scenario. Finally, a physiological increase in VAT/SAT ratio as well as an increase in adipose tissue density may be expected with increasing age [24]. Hence, adipose tissue characteristics may have a different effect on survival in different age ranges. This hypothesis could not be tested through stratified analyses in our cohort due to the small sample size. Nevertheless, the reported effect estimates are adjusted by age, sex, and stage.

Our study has certain strengths. Principally, it is the first study to assess the relationship between mortality and adipose tissue radiodensity in adult patients newly diagnosed with rectal cancer, a cancer with an increased incidence rate among young adults in Western countries [23]. Secondly, the inclusion of the vast majority of incident stage II–IV rectal cancers, which occurred in the resident population, i.e., approximately 83% of those reported by the local cancer registry, makes this study a population-based study, rather than a hospital-based cohort study. On the contrary, having cases from only one province, and all treated in different hospitals of the same cancer care network, reduces the generalizability of the results. Finally, adipose tissue parameters were collected using a CT scan, which is a routine imaging examination in rectal cancer staging and is considered a de facto gold standard method for evaluating body composition, especially for conducting abdominal body fat measurements in patients with rectal cancer [29,30]. The exclusion of stage I rectal cancer patients, who lacked a routine baseline staging CT scan, should not have led to a relevant loss of events, given the high survival of stage I patients, i.e., over 96% 5-year relative survival in our province [31]. However, some stage shift phenomenon can occur if patients are staged with different accuracy. This phenomenon, if present, could have resulted in a higher exclusion rate of older patients (less extensively staged at diagnosis), who also have physiologically altered adipose tissue characteristics. On the contrary, in younger patients, more cancers with good prognosis may be included in stage II, due to a more accurate local staging. Unfortunately, we do not have information on other factors, such as tumour grade, partly body mass index which is missing in almost 40% patients, and, more importantly, the changes in body weight prior to the cancer diagnosis (i.e., weight loss), that can be associated with disease severity at the onset (and, consequently, survival) and body composition. Furthermore, we lack information regarding lipid profile and general inflammatory markers. In fact, inflammation is one of the possible mechanisms of action behind the association between fat radiodensity and OS in cancer patients. Other important limitations are the relatively small sample size, which can affect the reliability of our findings, the retrospective design of the study, and the relatively short follow-up time. Owing to these limitations, our results are preliminary and need further replication. Finally, multicollinearity may have reduced the precision of estimates in models, including estimates of both density and area of fat compartments, areas which are strongly associated. However, in such models, the standard error of each SHR remained low, indicating precise estimates and thus allowing a reliable comparison with SHR obtained from models, including each measure alone (area or density).

Our finding may not have an immediate implication in a clinical setting; however, they raise awareness of the importance of a new dimension of variables (i.e., body fat radiodensity) that can be routinely measured in cancer patients, and which deserves more research in the field of oncology. The relatively strong association between fat density and CSS, in the absence of any association between fat density and PFS suggests, that the link is mostly due to non-oncologic factors acting on patient fragility. however, this hypothesis merits further investigation. If our findings are confirmed, consolidated and better understood, it will be of particular interest to develop cut-off points of adipose tissue radiodensity, capable of adding predictive value to known prognostic factors in rectal cancer patients. Finally, only by further clarifying the physiological and pathological meaning of adipose tissue radiodensity can we try to hypothesize focused interventions aimed at ameliorating this new biomarker.

## 5. Conclusions

In our study, we provide evidence which indicates that an increased radiodensity of abdominal body fat compartments at the time of diagnosis is associated with a higher risk of mortality in adult patients with rectal cancer. Undoubtedly, this finding needs to be further investigated in order to understand its underlying mechanisms and, eventually, to assess its usefulness in clinical practice.

## Figures and Tables

**Table 1:** Clinical characteristics and baseline CT adipose tissue parameters.

Clinical Characteristics	All Patients (*n* = 274)	Cancer-Specific Deaths (*n* = 116, 42.3%)	Progression or Death from Any Cause * (*n* = 63, 32.0%)
Age at diagnosis (years)	70 (59–79)	74 (61–81)	65 (52–76)
Female sex (%)	113 (41.2%)	48 (41.4%)	28 (44.4%)
**Stage**			
II	41 (15.0%)	11 (9.5%)	9 (14.3%)
III	161 (58.7%)	49 (42.2%)	54 (85.7%)
IV	72 (26.3%)	56 (48.3%)	
**BMI** (kg/m^2^)	21.2 (19.0–23.8)	20.3 (19.0–22.0)	20 (16.8–21.9)
<20	63 (23.0%)	25 (21.6%)	16 (25.4%)
20–24.9	75 (27.4%)	27 (23.3%)	18 (28.6%)
≥25	31 (11.3%)	5 (4.3%)	1 (1.6%)
Missing	105 (38.3%)	59 (50.9%)	28 (44.4%)
**Adipose tissue parameters**			
TAT area (cm^2^)	326 (219–445)	288 (205–432)	262 (167–417)
TAT density (HU)	−92 (−98–−85)	−91 (−96–−84)	−92 (−96–−85)
VAT area (cm^2^)	142 (84–221)	137 (77–216)	120 (55–177)
VAT density (HU)	−89 (−95–−80)	−87 (−94–−79)	−88 (−94–−78)
SAT area (cm^2^)	148 (103–204)	142 (99–189)	140 (78–186)
SAT density (HU)	−97 (−102–−91)	−95 (−102–−89)	−97 (−102–−91)
**Therapy**			
Neoadjuvant therapy (CT and/or RT)	135 (49.3%)	33 (28.5%)	43 (68.3%)
Surgery	181 (66.1%)	48 (41.4%)	57 (90.5%)
Adjuvant therapy (CT and/or RT)	117 (42.7%)	31 (26.7%)	37 (58.7%)
Palliative therapy (CT and/or RT)	60 (21.9%)	41 (35.3%)	3 (4.8%)
None	28 (10.2%)	23 (19.8%)	2 (3.2%)
Unknown	4 (1.5%)	2 (1.7%)	0

Data are reported as frequencies (percentage of total) for categorical variables and median (interquartile range) for continuous variables. * excluding stage IV or patients with missing data on progression. BMI, body mass index; TAT, total adipose tissue; VAT, visceral adipose tissue; SAT, subcutaneous adipose tissue; CT, chemotherapy; RT, radiotherapy.

**Table 2:** Competitive-risk analysis for cancer-specific survival (CSS) (including and excluding stage IV patients) and progression-free survival (PFS), adjusted for age, sex and stage.

	CSS					CSS (without Stage IV)					PFS				
	Pts	Events	SHR	95% CI	*p*	Pts	Events	SHR	95% CI	*p*	Pts	Events	SHR	95% CI	*p*
TAT I quartile (<219 cm^2^)	65	30	2.08	1.13–3.82	0.02	48	19	2.03	0.94–4.38	0.07					
TAT II quartile (221–326 cm^2^)	60	31	1.85	1.07–3.21	0.03	41	13	1.35	0.59–3.09	0.48					
TAT III quartile (327–445 cm^2^)	62	20	1 (ref)			47	11	1 (ref)							
TAT IV quartile (>445 cm^2^)	62	22	1.35	0.72–2.55	0.35	51	13	1.03	0.46–2.3	0.95					
TAT (one cm^2^ increase)											182	58	0.998	0.996–1.000	0.07
TAT density (one HU increase)	249	103	1.03	1.01–1.05	0.002	187	56	1.04	1.02–1.07	0.002	182	58	1.023	1.00–1.05	0.05
VAT (one cm^2^ increase)	255	108	0.998	0.996–1.001	0.160	191	59	0.998	0.995–1.000	0.072	186	60	0.997	0.994–1.000	0.07
VAT density (one HU increase)	252	108	1.02	1.002–1.038	0.027	188	59	1.025	1.003–1.047	0.023	183	60	1.014	0.993–1.035	0.20
SAT (one cm^2^ increase)	250	104	0.997	0.994–1.000	0.057	187	56	0.997	0.993–1.000	0.066	182	58	0.997	0.994–1.001	0.12
SAT density (one HU increase)	250	104	1.03	1.011–1.050	0.002	187	56	1.032	1.010–1.055	0.004	182	58	1.015	0.993–1.038	0.18
VAT/SAT ratio (one unit increase)	250	104	0.91	0.697–1.199	0.518	187	56	0.802	0.566–1.137	0.216	182	58	0.953	0.54–1.68	0.87

Pts, patients; SHR, sub-distribution hazard ratio; CI, confidence interval; TAT, total adipose tissue; VAT, visceral adipose tissue; SAT, subcutaneous adipose tissue.

**Table 3:** Competitive-risk analysis for cancer-specific survival (CSS) (including and excluding stage IV patients) and progression-free survival (PFS), adjusted for age, sex and stage, including each adipose tissue compartment area and density.

	CSS					CSS (without Stage IV)					PFS				
	Pts	Events	SHR	95% CI	*p*	Pts	Events	SHR	95% CI	*p*	Pts	Events	SHR	95% CI	*p*
**Model TAT**	249	103				187	56				182	58			
TAT I quartile (<219 cm^2^)			1.29	0.61–2.74	0.51			1.27	0.50–3.22	0.61					
TAT II quartile (221–326 cm^2^)			1.54	0.87–2.75	0.14			1.12	0.47–2.67	0.80					
TAT III quartile (327–445 cm^2^)			1 (ref)					1 (ref)							
TAT IV quartile (>445 cm^2^)			1.63	0.84–3.16	0.15			1.29	0.56–2.99	0.56					
TAT (one cm^2^ increase)													0.999	0.996–1.001	0.27
TAT density (one HU increase)			1.03	1.00–1.07	0.03			1.04	1.00–1.07	0.03			1.007	0.973–1.043	0.69
**Model VAT**	252	108				188	59				183	60			
VAT (one cm^2^ increase)			1.00	0.997–1.003	0.82			0.999	0.996–1.003	0.66			0.997	0.992–1.001	0.17
VAT density (one HU increase)			1.022	0.996–1.050	0.10			1.020	0.990–1.052	0.20			0.998	0.969–1.028	0.89
**Model SAT**	250	104				187	56				182	58			
SAT (one cm^2^ increase)			1.000	0.997–1.003	0.95			0.999	0.996–1.003	0.67			0.997	0.993–1.001	0.21
SAT density (one HU increase)			1.029	1.003–1.056	0.03			1.029	1.000–1.058	0.05			1.002	0.974–1.031	0.89

Pts, patients; SHR, sub-distribution hazard ratio; CI, confidence interval; TAT, total adipose tissue; VAT, visceral adipose tissue; SAT, subcutaneous adipose tissue.

## Data Availability

According to Italian law, anonymized data can only be made publicly available if there is potential for the re-identification of individuals (https://www.garanteprivacy.it, accessed on 15 December 2022). Furthermore, the ownership of the data is that of the patient, who gave consent for these data to be used for the objective of the study. Thus, the data underlying this study are available on request for researchers intending to conduct research and respect confidentiality (even if anonymous data are provided, they should be published in aggregated form) in studies with objectives consistent with those of the original study. In order to obtain the data, approval must be obtained from the Area Vasta Emilia Nord (AVEN) Ethics Committee, who will check the consistency of the objective and planned analyses and will then authorize us to provide aggregated or anonymized data. Data access requests should be addressed to the Ethics Committee at CEReggioemilia@ausl.re.it, as well as to the authors at the Epidemiology Unit of AUSL-IRCCS in Reggio Emilia at info.epi@ausl.re.it, who are the data guardians.

## Supplementary Materials

The following supporting information can be downloaded at: https://www.mdpi.com/article/10.3390/nu15020374/s1. Figure S1. Semi−automated measurement of TAT, VAT, and SAT areas and densities on unenhanced CT images. Figure S2. Kaplan−Meier survival curves for OS and PFS by quartiles of VAT area and VAT density. Figure S3. Kaplan−Meier survival curves for OS and PFS by quartiles of SAT area and SAT density. Figure S4. Scatter plot of each adipose compartment area by its respective density. Figure S5. Scatter plot of VAT density by SAT density. Table S1. Distribution of adipose tissue characteristics according to stage. Table S2. Distribution of adipose tissue characteristics according to age and sex. Table S3. Preliminary analysis to verify the linearity of the association between CT adipose tissue parameters (quartiles) and survival, by coefficients obtained by means of an unadjusted Cox proportional hazards model. Table S4. Competitive−risk analysis including bot SAT and VAT density for CSS (including and excluding stage IV patients) and PFS, adjusted for age, sex and stage.
