# Supplementary material for: Abdominal Fat Characteristics and Mortality in Rectal Cancer: A Retrospective Study
Source: Nutrients. 2023 Jan 11;15(2):374. doi: 10.3390/nu15020374 (PMC9864407; doi:10.3390/nu15020374)
Supplement: Supplementary file 1 [file nutrients-15-00374-s001.zip › nutrients-2137738-supplementary.pdf]

nutrients

MDPI

Supplementary Materials

Abdominal Fat Characteristics and Mortality in Rectal Cancer: A Retrospective Study

Massimo Pellegrini †, Giulia Besutti *,†, Marta Ottone, Simone Canovi, Efrem Bonelli, Francesco Venturelli, Roberto Farì, Angela Damato, Candida Bonelli, Carmine Pinto, Guido Ligabue, Pierpaolo Pattacini, Paolo Giorgi Rossi and Marwan El Ghoch

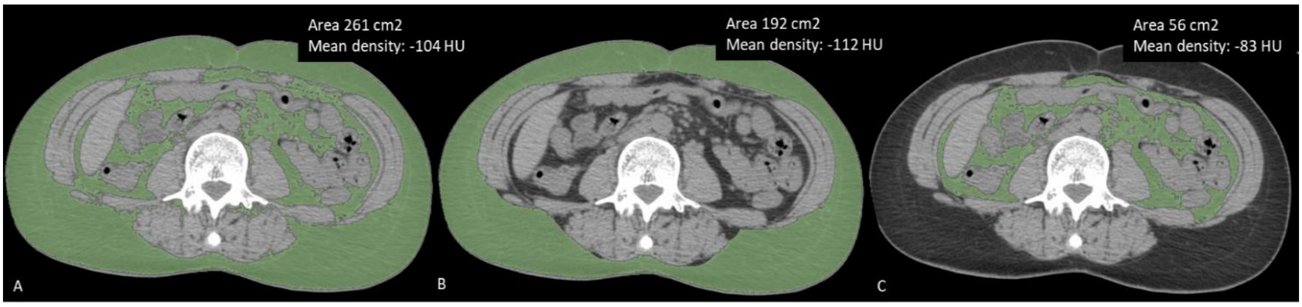

Figure S1. Semi–automated measurement of TAT, VAT, and SAT areas and densities on unenhanced CT images.

Table S1. Distribution of adipose tissue characteristics according to stage.

	Stage			
	II	III	IV	*p*
TAT area	374 (262 to 553)	326 (205 to 459)	292 (207 to 399)	0.0799
TAT density	−95 (−99 to −88)	−92 (−98 to −85)	−91 (−97 to −83)	0.1570
VAT area	171 (105 to 263)	137 (81 to 219)	132 (81 to 198)	0.1293
VAT density	−93 (−97 to −86)	−89 (−95 to −79)	−84 (−94 to −79)	0.0221
SAT area	157 (131 to 188)	144 (101 to 211)	145 (97 to 203)	0.5166
SAT density	−98 (−103 to −93)	−97 (−102 to −91)	−97 (−102 to −88)	0.4439
VAT/SAT	1.20 (0.70 to 1.93)	0.82 (0.49 to 1.35)	0.96 (0.65 to 1.34)	0.0606

TAT, total adipose tissue; VAT, visceral adipose tissue; SAT, subcutaneous adipose tissue.

Table S2. Distribution of adipose tissue characteristics according to age and sex.

	Age			Sex		
	<65 years	≥65 years	*p*	Male	Female	*p*
TAT area (cm²)	308 (190 to 418)	359 (234 to 479)	0.041	360 (221 to 465)	289 (209 to 412)	0.066
TAT density (HU)	−94 (−99 to −89)	−91 (−97 to −84)	0.009	−92 (−98 to −84)	−93 (−98 to −87)	0.431
VAT area (cm²)	120 (58 to 170)	160 (100 to 255)	<0.001	168 (103 to 253)	113 (54 to 160)	<0.001
VAT density (HU)	−88 (−93 to −81)	−90 (−97 to −79)	0.188	−91 (−97 to −81)	−87 (−94 to −77)	0.005
SAT area (cm²)	154 (109 to 239)	144 (102 to 188)	0.181	141 (96 to 185)	168 (113 to 237)	0.006
SAT density (HU)	−98 (−104 to −93)	−95 (−100 to −90)	0.003	−96 (−101 to −89)	−98 (−103 to −93)	0.011
VAT/SAT	0.71 (0.41 to 1.01)	1.15 (0.74 to 1.63)	<0.001	1.23 (0.83 to 1.78)	0.63 (0.38 to 0.80	<0.001

TAT, total adipose tissue; VAT, visceral adipose tissue; SAT, subcutaneous adipose tissue.

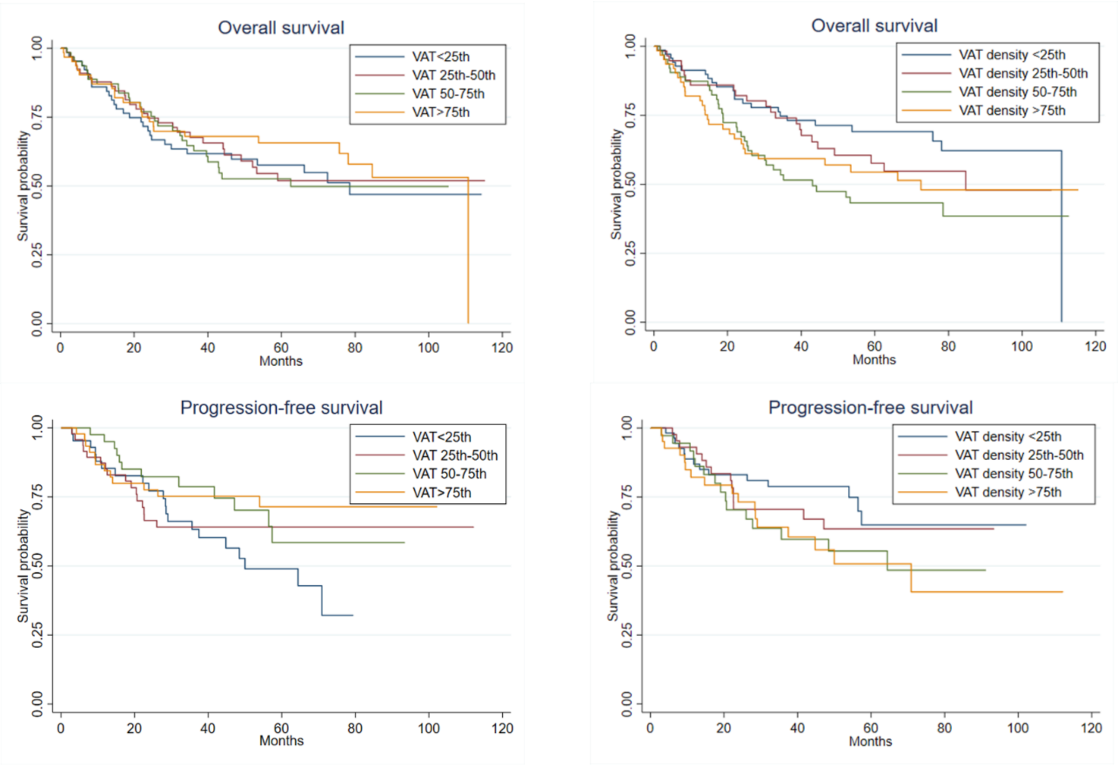

Figure S2. Kaplan–Meier survival curves for OS and PFS by quartiles of VAT area and VAT density.

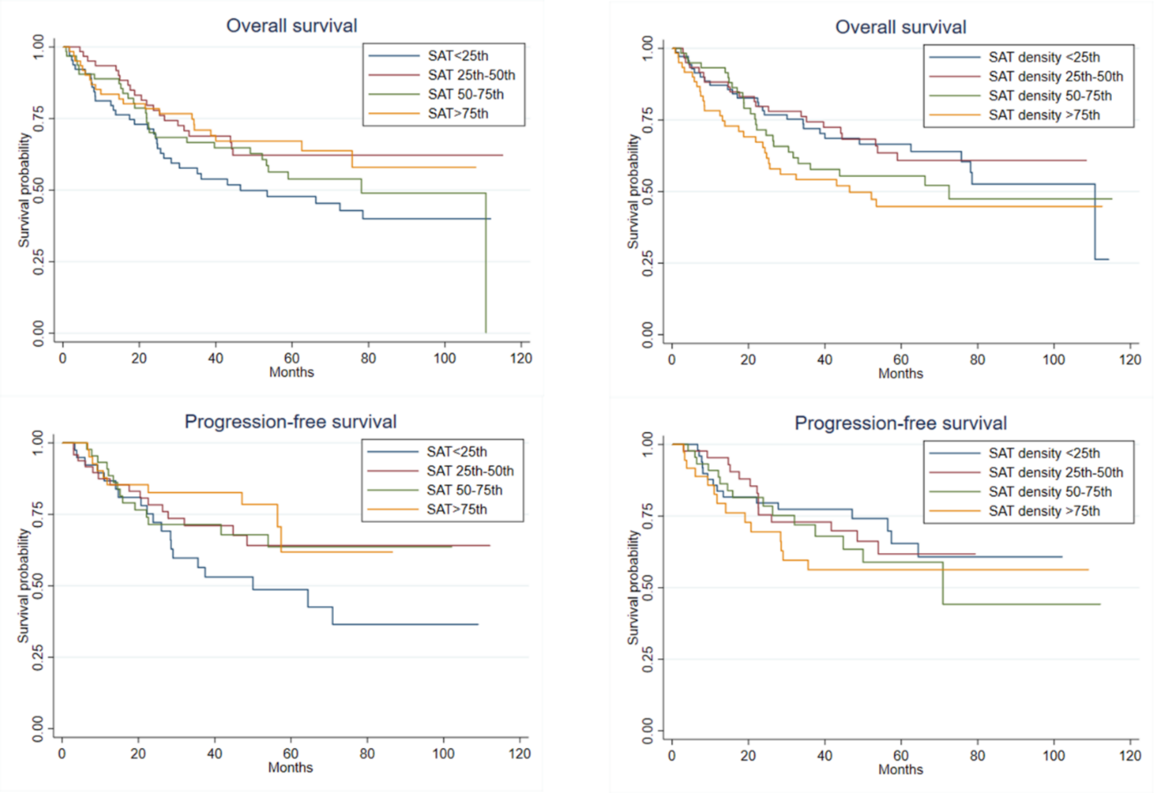

Figure S3. Kaplan–Meier survival curves for OS and PFS by quartiles of SAT area and SAT density.

Table S3. Preliminary analysis to verify the linearity of the association between CT adipose tissue parameters (quartiles) and survival, by coefficients obtained by means of an unadjusted Cox proportional hazards model.

	Overall survival	Progression−free survival
	β (95%CI)	β (95%CI)
TAT		
I quart [<221]		
II quart [221−326]	0.152 (−0.314; 0.618)	−0.703 (−1.384; −0.022)
III quart [327−445]	−0.274 (−0.777; 0.229)	−0.681 (−1.362; 0.000)
IV quart [>445]	−0.133 (−0.621; 0.355)	−1.016 (−1.788; −0.245)
TAT density		
I quart [<−98]		
II quart [−98 − −92]	0.084 (−0.451; 0.618)	0.130 (−0.638; 0.899)
III quart [−93 − −85]	0.681 (0.182; 1.181)	0.463 (−0.281; 1.207)
IV quart [>−85]	0.723 (0.218; 1.229)	0.624 (−0.099; 1.347)
VAT		
I quart [<84]		
II quart [84−142]	−0.634 (−0.543; 0.416)	−0.196 (−0.854; 0.462)
III quart [143−221]	0.042 (−0.434; 0.517)	−0.451 (−1.150; 0.247)
IV quart [>221]	−0.064 (−0.547; 0.419)	−0.656 (−1.392; 0.080)
VAT density		
I quart [<−95]		
II quart [−95 − −89]	0.301 (−0.210; 0.812)	0.242 (−0.562; 1.045)
III quart [−88 − −80]	0.554 (0.070; 1.039)	0.657 (−0.087; 1.400)
IV quart [>−80]	0.527 (0.037; 1.017)	0.729 (−0.030; 1.488)
SAT		
I quart [<103]		
II quart [103−148]	−0.432 (−0.915; 0.052)	−0.590 (−1.276; 0.095)
III quart [148−204]	−0.172 (−0.626; 0.283)	−0.495 (−1.181; 0.191)
IV quart [>204]	−0.444 (−0.939; 0.051)	−0.783 (−1.532; −0.034)
SAT density		
I quart [<−102]		
II quart [−102 − −97]	−0.064 (−0.592; 0.463)	0.076 (−0.653; 0.805)
III quart [−96 − −91]	0.440 (−0.038; 0.918)	0.269 (−0.449; 0.986)
IV quart [>−91]	0.481 (−0.000; 0.962)	0.445 (−0.284; 1.173)

β coefficients with respective 95% confidence intervals are reported.

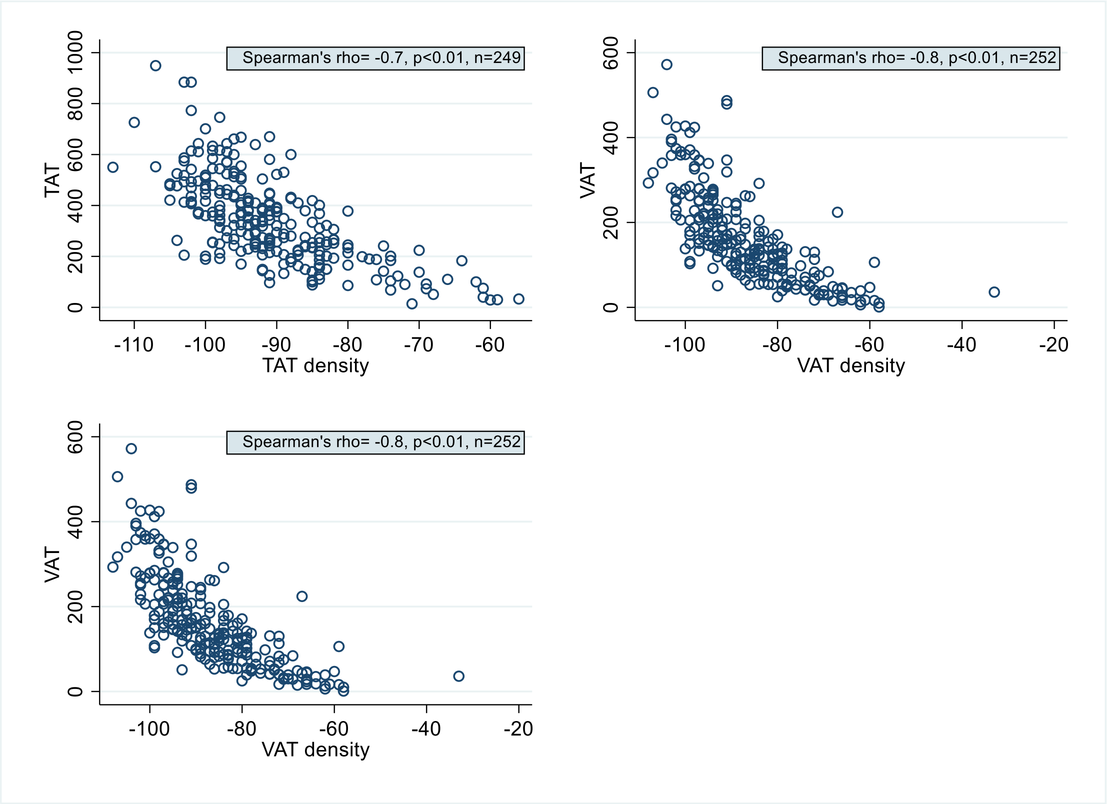

Figure S4. Scatter plot of each adipose compartment area by its respective density.

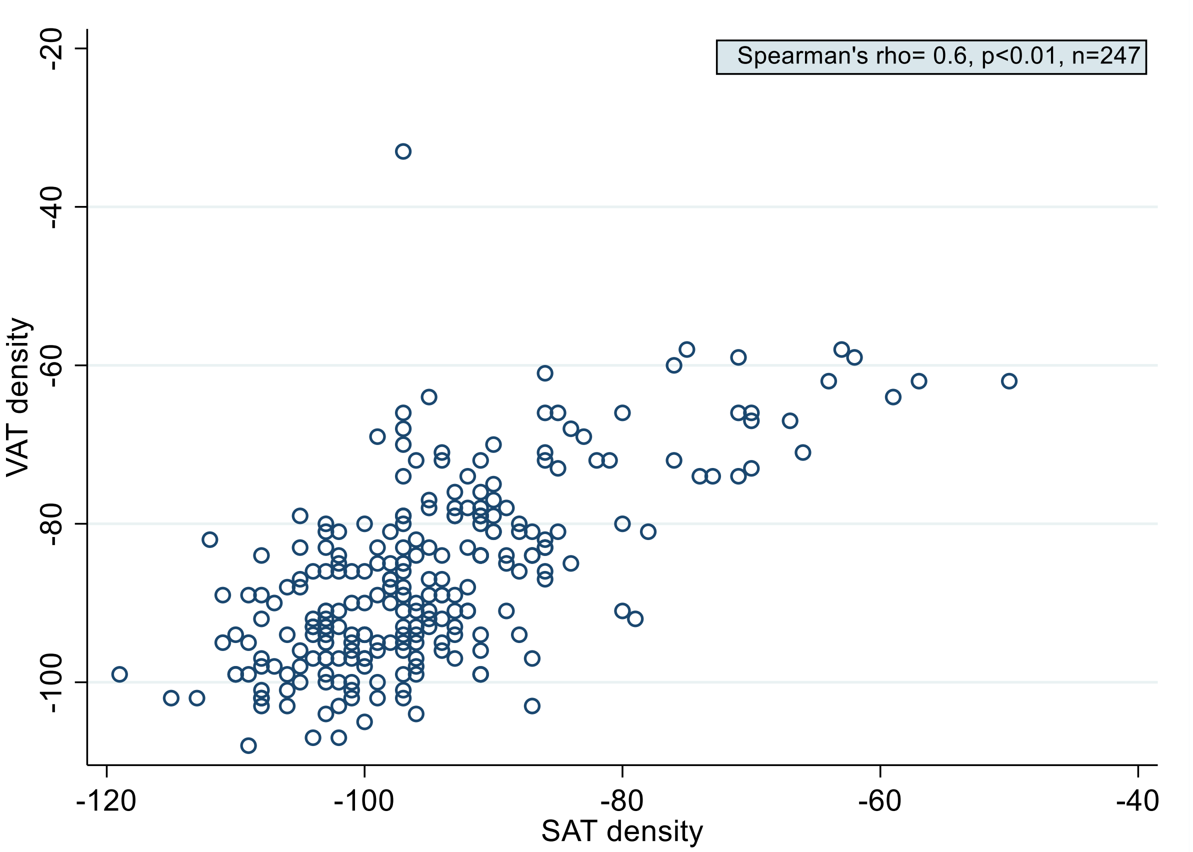

Figure S5. Scatter plot of VAT density by SAT density.

Table S4. Competitive−risk analysis including bot SAT and VAT density for CSS (including and excluding stage IV patients) and PFS, adjusted for age, sex and stage.

	**CSS**			**CSS (without stage IV)**			**PFS**		
**Models including both SAT and VAT density**	SHR	95%CI	*p*	SHR	95%CI	*p*	SHR	95%CI	*p*
VAT density (one HU increase)	1.006	0.983–1.030	*0.601*	1.015	0.988–1.043	*0.286*	1.011	0.985–1.037	*0.423*
SAT density (one HU increase)	1.024	0.998–1.051	*0.075*	1.019	0.988–1.051	*0.228*	1.005	0.976–1.036	*0.724*
